# Protocol for image-based small-molecule screen to identify neuroprotective compounds for dopaminergic neurons in zebrafish

**DOI:** 10.1016/j.xpro.2024.102837

**Published:** 2024-01-12

**Authors:** Gha-hyun Jeffrey Kim, Min Chen, Sharie Kwok, Su Guo

**Affiliations:** 1 Department of Bioengineering and Therapeutic Sciences, University of California, San Francisco, San Francisco, CA, USA; 2 School of Pharmacy, University of California, San Francisco, San Francisco, CA, USA

**Keywords:** High-Throughput Screening, Model Organisms, Neuroscience

## Abstract

Whole-organism-based screen holds promise for discovering biologically active compounds. However, high-content imaging is challenging due to the difficulty of positioning live animals and individual variability of neuron counts. Here, we present a protocol to identify neuroprotective compounds for dopaminergic neurons in zebrafish using an image-based small-molecule screen. We describe steps for raising larvae, agarose embedding, and treatment to induce neurodegeneration. We then detail procedures for live confocal imaging, image processing, and data analysis.

For complete details on the use and execution of this protocol, please refer to Kim et al. (2021).^1^

## Highlights

• Whole-organism *in vivo* imaging-based screening protocol for neurodegenerative diseases
• Step-by-step guide to embed and position larvae dorsal down in 96-well plates
• Live confocal imaging before and after treatment of zebrafish larvae diencephalon
• Automated pipeline for quantifying dopamine neuron loss with open-source program

## Abstract (editor highlights)

Publisher’s note: Undertaking any experimental protocol requires adherence to local institutional guidelines for laboratory safety and ethics.

## Graphical abstract

**Figure:**
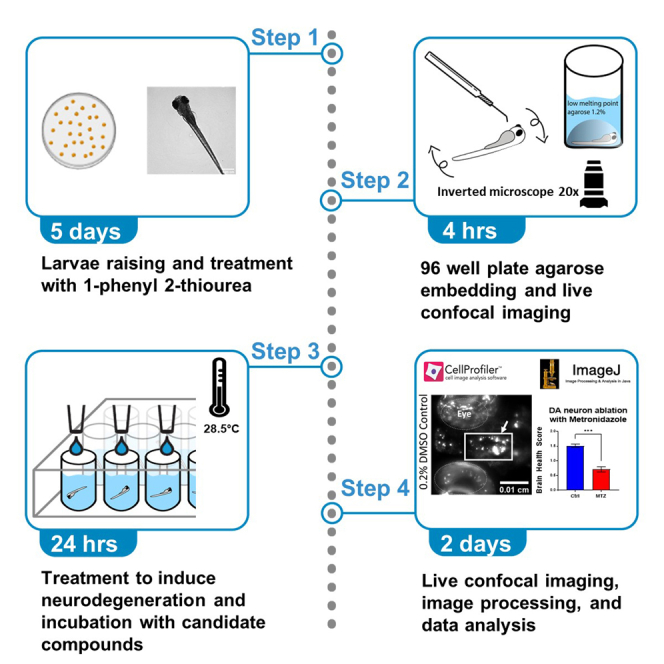

## Before you begin

The protocol below describes an *in vivo* confocal imaging-based method to identify neuroprotective small molecules for dopaminergic neurons in larval zebrafish. Before starting the experiment, researchers should have the appropriate zebrafish with expression of the bacterial enzyme nitroreductase (NTR) fused with a fluorescent reporter (e.g., mCherry) driven by the tyrosine hydroxylase (th) promoter. The library selection and solubility of candidate compounds should also be reviewed by literature to ensure all control and test samples are treated with the same DMSO concentration.

### Zebrafish husbandry and transgenic lines

**Timing: 3 months**

Here we will describe the selection and breeding of homozygous Tg[fuguth:gal4-uas:GFP; uas-NTRmCherry] lines that will be used for the assay. Having a sufficient batch of homozygous transgenic adult zebrafish expressing Tg[fuguth:gal4-uas:GFP; uas-NTRmCherry] is important as it allows for a consistent production of embryos and creating a weekly protocol for high throughput drug screening.

**Figure 1:**
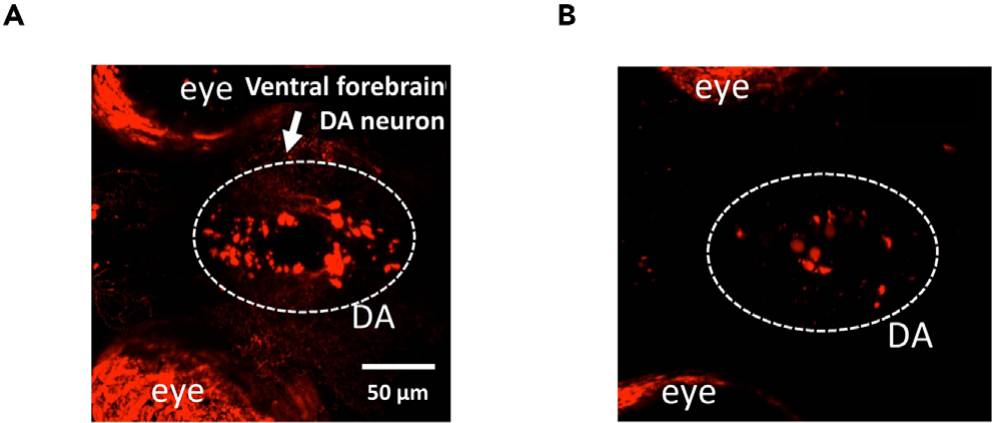
Th-NTR-mCherry expression in larvae (A) Example of a potentially homozygous larvae showing very bright fluorescence under the microscope as compared to (B) weak signals which are indicative of heterozygous lines. Sort the larvae that express very bright red fluorescence.

1. Generate homozygous Tg[fuguth:gal4-uas:GFP; uas-NTRmCherry] lines.
  a. Cross two heterozygous Tg[fuguth:gal4-uas:GFP; uas-NTRmCherry] adult zebrafish to obtain embryos.
  b. On day 4 or 5, screen larvae under a fluorescence stereo microscope with GFP and RFP.
    i. To temporarily immobilize the larvae during screening, treat the larvae with tricaine at a low concentration (final concentration 160 μg/mL) 30 min prior to screening.
    ii. Sort the larvae that show strong fluorescence under RFP and label as “potentially homozygous”.
2. Raise the potentially homozygous Tg[fuguth:gal4-uas:GFP; uas-NTRmCherry]
3. Identify homozygous lines.
  a. Cross one potentially homozygous Tg[fuguth:gal4-uas:GFP; uas-NTRmCherry] larvae with AB WT.
  b. Between 3 dpf and 5 dpf, screen larvae under the fluorescent RFP.
  c. If all larvae express red TH neurons as shown in Figure 1, the parent fish is considered to be homozygous and can be utilized as breeders for screening.

**CRITICAL:** To ensure proper rearing of zebrafish to adulthood with optimal light-dark cycles, feeding methods for different stages, and handling of fish tanks, refer to chapter 3 of The Zebrafish Book published by the ZFIN guidelines.^2^

### Institutional permissions

The zebrafish is a vertebrate species. The study was reviewed and approved by the University of California, San Francisco Institutional Animal Care and Use Committee (approval number AN179000). The zebrafish system was regularly inspected by the University of California, San Francisco Laboratory Animal Resource Committee.

## Key resources table

**Table:** 

REAGENT or RESOURCE	SOURCE	IDENTIFIER
**Biological samples**		

*Danio rerio*, AB wild type	Zebrafish International Resource Center	ZFIN ID: ZDB-GENO-960809–7
*Danio rerio*, fuguth:gal4-uas: GFP; uas-NTRmCherry	https://doi.org/10.1371/journal.pone.0164645	UCSF Guo Lab
*Danio rerio*, th1:gal4; uas:NTRmCherry	https://doi.org/10.1016/j.nbd.2016.07.020	Jiulin Du lab

**Chemicals, peptides, and recombinant proteins**		

Bioactive Compound Library (1,403 compounds)	SelleckChem; curated set from UCSF Small Molecular Discovery Center. This is an example library	Cat# L1700
Metronidazole (MTZ)	Selleck Chemicals	Cat# S1907
Conduritol B epoxide (CBE)	Sigma-Aldrich	Cat# 6090-95-5
Low melting point agarose	IBI	IB70050
Tricaine	Sigma-Aldrich	Cat# A5040-100 *g*
1-Phenyl 2-thiourea (PTU)	Sigma-Aldrich	Cat# P7629
DMSO, anhydrous	G-Biosciences	Cat# BKC-17
Methylene blue	MilliporeSigma	Cat #M9140
CaSO_4_	MilliporeSigma	Cat# 255696

**Deposited data**		

IN Cell Analyzer 6000 acquisition protocol (20x objective)	https://doi.org/10.5281/zenodo.10360186	https://github.com/happyiowa/screening-protocol
Custom CellProfiler pipeline for image analysis	https://doi.org/10.5281/zenodo.10360186	https://github.com/happyiowa/screening-protocol

**Software and algorithms**		

ImageJ	NIH	RRID: SCR_003070
Prism 7	GraphPad	RRID: SCR_002798
MATLAB	MathWorks	RRID: SCR_001622
CellProfiler (v4.2)^3^	The Broad Institute of Harvard and MIT	RRID: SCR_007358
IN Cell Analyzer	GE Life Sciences	RRID: SCR_015790

**Others**		

Flat bottom 96-well plate (Greiner μClear)	Greiner	Cat# 655096
LED light pad (optional for better plate visualization)	HSK	Cat# B079HMW4BV
Probe 0.25 mm tip	World Precision Instruments	Cat# WPI0118
Tube heat block	Techne	Cat# 1200T14

**CRITICAL:** Metronidazole in aqueous solution is sensitive to sunlight and UV radiation so ensure it is stored in a dry cool area with minimal light. When handling metronidazole, ensure fitting proper protective equipment including gloves and safety goggles. Conduritol B Epoxide is considered nonhazardous but the aqueous formulation should only be used within a day so always prepare a fresh aqueous batch for each experiment.

The reagents were obtained from the specified manufacturers in the key resources table but these can be substituted with any other manufacturer. However, it is important to ensure the exact reagents are being used. i.e., metronidazole has a molecular weight of 171.16 *g*/mol. There are other compounded products such as metronidazole benzoate which has different properties and may not be suitable for this assay.

The tricaine used as the anesthetic for embedding should not be substituted with other anesthetics such as isoflurane and lidocaine. For the 96 well plate, a flat bottom plate is highly recommended as other shapes such as the round bottom made larvae positioning more difficult and required more agarose volume.

## Materials and equipment

**Table:** Blue Egg Water (BEW)

Reagent	Final concentration	Amount
NaCl	15 mM	9.0 *g*
CaSO_4_	8.3 mM	2.25 mL
Methylene blue (2.303%)	2.303%	100 μL
ddH_2_O	N/A	Fill up to 10 L
**Total**	**N/A**	**10 L**

BEW can be stored between 20°C–25°C. Avoid using BEW that has been more than 2 weeks. Prepare fresh BEW if there is apparent algae growth or loss in the blue tint.

**Table:** 45 mM Metronidazole stock and 96-well plate dilution

Reagent	Final concentration	Amount
Metronidazole	45 mM	7.7 *g*
DMSO	1%	10 mL
Blue Egg water	N/A	990 mL
**Total**	**N/A**	**1 L**

It is advised to aliquot the stock in 50 mL falcon tubes and store in ‒20°C. Avoid using MTZ stock if stored more than 6 months. Do a 5 fold dilution for the experiment to achieve a final concentration of 9 mM MTZ with 0.2% DMSO that is used for the 96 well screening plate.

**Table:** 1.2% low melting point agarose (LMP) w/ low dose tricaine

Reagent	Final concentration	Amount
LMP agarose	1.2%	0.24 *g*
Low dose tricaine	160 μg/mL	3.2 *g*
Milli-Q water	N/A	Up to 20 mL
**Total**	**N/A**	**20 mL**

The LMP agarose should be aliquoted into 2 mL Eppendorf tubes and maintained in the 50°C heat block. Always make fresh agarose for the weekly experiments.

Tricaine stock preparation: 4 mg/mL; weigh 400 mg tricaine and dissolve in 97.1 mL ddH_2_O. Add 2.9 mL of 1 M Tris-HCl 9.0, to adjust the PH to ∼7.5. For general use, dilute 1:25 to the working concentration of 160 μg/mL. For low dose tricaine treatment (e.g., 24 h incubation in agarose), dilute 1:100 to the working concentration of 40 μg/mL.

**CRITICAL:** the LMP agarose and tricaine should be prepared exactly as described in the protocol. These concentrations were determined based on different experiments and were determined to be the best conditions to ensure the larvae are in stable conditions during imaging and the 24 hour incubation period.

PTU stock preparation: Weigh 0.3 *g* of PTU and add to 100 mL ddH_2_O to make a suspension stock of 0.3%. Shake the bottle well prior to use. Dilute 1:100 to the working concentration of 0.003%.

**CRITICAL:** PTU can be harmful if swallowed, inhaled, or absorbed through the skin. It may cause respiratory and skin irritation. Always prepare stock solution with gloves in a well-ventilated area.

## Step-by-step method details

### Collect and raise transgenic embryos for screening

**Timing: 5 days**

This section describes the process of breeding zebrafish to produce sufficient larvae for 5 dpf and 6 dpf screening. The protocol is designed in a way that can be a routine screening assay that can be conducted on a weekly basis. By setting up zebrafish crosses midweek, while conducting the chemical treatment for the current batch, the experimenter(s) can be do subsequent compound screenings or validation studies without any gaps or delays.

1. In the late afternoon or early evening, set up homozygous Tg[fuguth:gal4-uas:GFP; uas-NTRmCherry] crosses with AB Wild Type, by putting one female and one male fish in a tank separated by a divider.

(optional) For obtaining a large number of eggs, pairing a female to male ratio of 2:1 during the crossing has shown to be safe and effective.

2. The next morning, pull the divider. Wait for 1–2 h. Collect embryos (Day 0) and transfer them to 30 mL of Blue Egg Water (2.4 *g* CaSO_4_, 4 *g* IO Salt, 600 μL of 1% Methylene per 20 L) in a Petri dish.
3. Raise the larvae in an incubator with temperatures set at 28.5°C.
4. At 24 hpf (hours post fertilization), inspect the embryos and remove unfertilized eggs.
5. Treat the remaining larvae with 0.003% of 1-phenyl 2-thiourea and place back in the incubator.
6. On 4 dpf (days post fertilization), observe the mCherry^+^ TH neurons under the fluorescence stereo microscope RFP channel.
7. Transfer the mCherry^+^ larvae into a fresh BEW Petri dish and treat with PTU.
8. Place the larvae back into the incubator.

***Note:*** Ensure standard light dark cycle of 14/10 h for crossing. When collecting embryos, ensure there are no more than 40 embryos per Petri dish with Blue Egg Water (BEW) during raising.

### Agarose embedding in 96-well plate

**Timing: 1–3 h per 96-well plate**

The 96 well plate embedding is important as proper positioning of the larvae translates to higher image quality and analysis. Here we detailed the steps to ensure the best practice technique for proper embedding.

9. Prepare 1.2% low melting point (LMP) agarose in BEW. Make 2 mL aliquots in Eppendorf tubes and place them in a 50°C heat block.
10. For each agarose tube to be used for embedding, add 20 μL of 4 mg/mL tricaine stock to each. Approximately 3 aliquot tubes are alternately used for a single 96 well plate.
11. Prepare 200 μL pipettes and cut the pipette tips to ∼3 mm opening, enough to ensure the larvae can go through.

**CRITICAL:** the pipette tips should be cut in a manner that is just enough for the larvae to move without being damaged. Having too large of a diameter (cutting too high in the pipette) will not only make it difficult for pipetting, but also inaccurate volume.

12. Treat the Petri dish containing 5 dpf Tg[fuguth:gal4-uas:GFP; uas-NTRmCherry] larvae with 800 μL of tricaine stock. Wait 5 min.
13. Use the 200 μL micropipette and gently draw the anesthetized larvae by setting the micropipette to 40 μL and transfer it to the agarose tube.

***Note:*** Minimize the amount of BEW used during the transfer.

14. After submerging the larvae in the agarose tube, transfer the larvae from the agarose tube to the 96 well plate.

***Note:***Methods video S1 describes the flow of steps 5 and 6, from pipetting the anesthetized larvae, submerging the larvae in agarose, and transferring to 96 well plate.

15. Carefully position the larvae in a dorsal down position (for an inverted microscope) using forceps. Let the agarose solidify.

**CRITICAL:** embedding the larvae in the correct position is important because it can significantly affect the image quality. The goal is to position the larvae dorsally closest to the lens. In this protocol, since the confocal microscope is inverted, the larvae are embedded dorsal down.

***Note:*** Sometimes positioning the larvae immediately after transferring to the 96 well plate can be difficult due to the viscosity of the agarose. Letting the larvae sit in the well for about 1 minute can allow for easier embedding. For best efficiency, transferring ∼5 larvae at a time and embedding them can be a good way to minimize delays while giving enough time for the agarose to solidify. Methods video S2 describes the careful adjusting of the larvae position using the probes.

Methods video S1. Transferring larvae from agarose to 96-well plate, related to steps 5 and 6 Starting from anesthetized larvae in the petri dish, a 200 μL pipette is used to transfer the larvae to the 1.2% LMP agarose tube. After submerging the larvae in agarose, the larvae is then transported to a single well in the 96 well plate.

**Figure 2:**
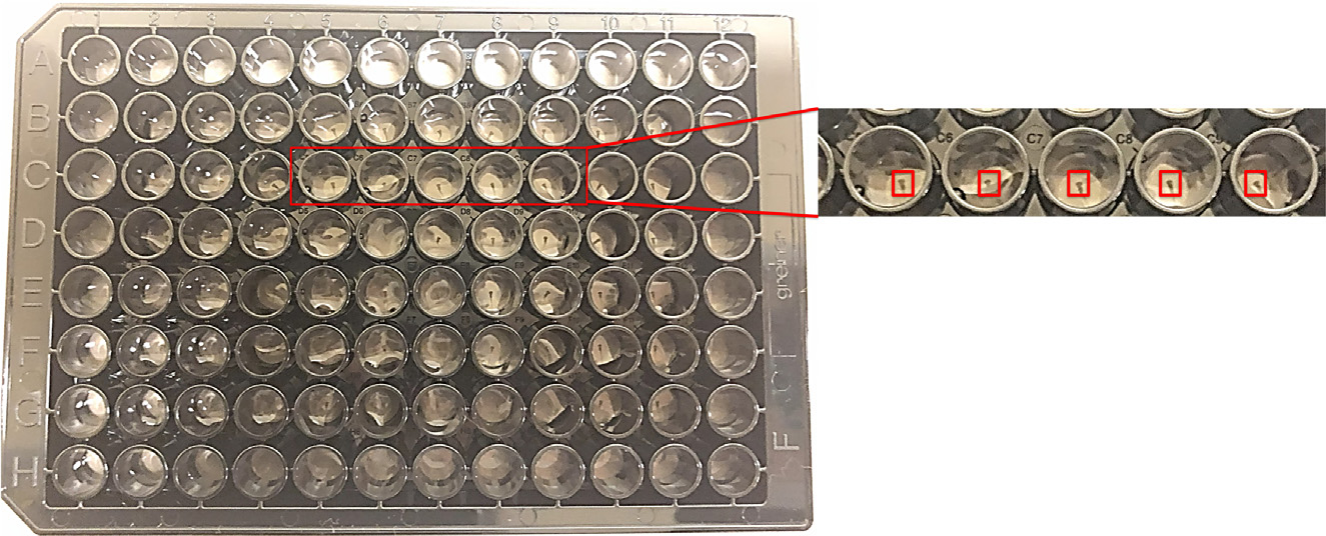
Embedding larvae in the 96-well plate A single 5 dpf larvae is embedded per well with 40 μL of low melting point agarose. The zoomed in section shows each larvae is correctly positioned in the center of the well. The red square represents the estimated area that the 20x confocal lens field of view.

***Note:*** The 40 μL agarose was chosen based on a previous experiment performed to optimize the amount of agarose to BEW ratio when embedding. 40 μL was determined to be the optimal balance between ease of embedding while not harming or stressing larvae during the 24 h incubation period. As this volume is plate specific, it is advised to test different agarose volume.

16. After embedding the larvae in the wells, add 100 μL BEW (no tricaine) to each well using a multichannel pipette. An example of the embedded larvae is shown in Figure 2.

Methods video S2. 96-well plate embedding of the larvae in the dorsal down position, related to step 7 Using a probe, the larvae is gently positioned in the dorsal down position. It is important to avoid direct contact with the larvae and use the viscosity of the agarose to maneuver the head and trunk for proper positioning.

### Confocal imaging and compound treatment

**Timing: 2 h for 2 consecutive days**

The whole organism based phenotypic drug discovery method described in this protocol is a powerful method that focuses on the direct impact on neuronal integrity. The imaging consists of two sessions pretreatment and post treatment with compounds. By imaging the larvae twice, this significantly reduces the intravariability of DA neurons. This section describes the example plate design and detailed process of the imaging and chemical treatment.

17. Place the agarose embedded 96 well plate in the IN Cell Analyzer 6000 and close the lid.
18. Determine the configurations of the 96 well plate (thickness, material, brand, shape).

**CRITICAL:** What plate you use can significantly affect the quality of the image so it is important to ensure the same plate is used across a screening batch. The Greiner μClear flat bottom plates were selected for our experiment as it provided a good surface area for embedding and image quality.

19. Use the 20x objective lens with the bright-field and DsRed fluorescence. Arrange the field of view toward the diencephalon region of the brain.
20. Observe a few wells and create a stack spanning ∼100 μm to cover the diencephalon region of the brain. Set each slice to 3 μm. Set the capture mode as “max intensity” projection.

***Note:*** This step is to create a stack of slices to ensure all 96 wells are covered.

21. For future use, save the settings within the .xaqp file. We have shared the .xaqp acquisition protocol in the GitHub repository https://github.com/happyiowa/screening-protocol (https://doi.org/10.5281/zenodo.10360186).

***Note:*** The parameters used for the imaging protocol including the z-stack thickness and exposure will vary depending on what device is being used. We used the IN Cell Analyzer 6000 (RRID: SCR_015790) and the .xaqp file contains all the preset parameters used for this experiment. The experimenter should ensure the same parameter settings are used for all plates for consistency and to minimize batch effects.

22. After imaging, remove the plate from the IN Cell Analyzer.
  a. Prepare a fresh batch of 45 mM MTZ in BEW (or 100 mM Conduritol B epoxide in 100% DMSO), and 10 mM of the screening compounds in 100% DMSO.
  b. Calculate the volume needed to achieve the desired final concentrations.

***Note:*** Our protocol is based on the following final concentrations: 9 mM MTZ, 500 μM CBE, and 10 μM of the screening compound.

23. Treat the 96 wells with MTZ (or CBE), and the screening compounds based on the plate setup.

**Figure 3:**
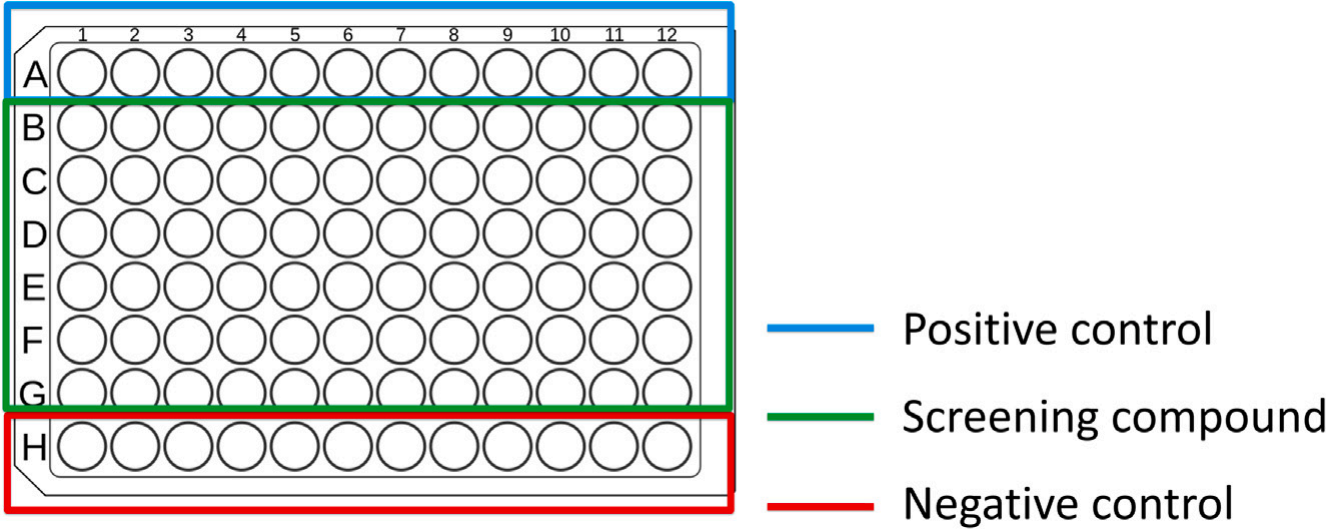
Example 96 well plate setup Each row represents as a single condition (n = 12). The top and bottom row are considered positive (0.2% DMSO), and negative control (MTZ or CBE) with screening compounds + MTZ/CBE in the middle rows.

***Note:*** To ensure adequate sample size, it is recommended to use one condition for each row (n = 12). Sample plate conditions are as follows: Top row positive control (0.2% DMSO), bottom row negative control (9 mM MTZ or 500 μM CBE), and the screening compounds + MTZ or CBE in between. An example of the plate setup is shown in Figure 3. The optional light box can be useful during this step to clearly visualize the chemical treatment. Place the lightbox under the 96 well plate and inspect for any chemical impurities or misplaced larvae.

### Image analysis

**Timing: 5 h**

The large dataset of confocal images obtained are analyzed with the CellProfiler image analysis software. The software automatically identifies the DA neurons by using the bright-field images of the larvae brain and quantifies the intensity. All information on the custom pipeline are shared in the repository.

24. The video explaining the detailed analysis pipeline can be found in the GitHub repository https://github.com/happyiowa/screening-protocol.
25. The open source CellProfiler image analysis software by the Broad Institute was used for analyzing the diencephalon DA neurons of zebrafish.
26. Download the latest CellProfiler for MacOS or Windows in https://cellprofiler.org/releases. The version used for this analysis was on version 4.2.
27. The image files or folders of the images can be uploaded directly to CellProfiler in the “images” tab.
28. Go to the “NamesAndTypes” section and click “update” to assign all images with the corresponding image type, fluorescence, and designated name.

***Note:*** If there is a mismatch in the rules or names assigned, the images will not be updated. Fix any matching rules if this occurs as this step is required to proceed with the pipeline.

29. Click on the “Smooth” function tab. The bright field images are used as a mask to locate the approximate position of the diencephalon.Select the bright-field images as the input.
30. Use the “Gaussian Filter” to blur and obscure features smaller than the specified diameter.
31. Next, proceed to the “ImageMath” tool to invert the 8-bit fluorescence image and propagate the image to identify DA neurons. Apply a 1.0 multiplication operation for the image inversion.
32. After the bright-field images have been smoothed and inverted, move to the “MaskImage” tab to overlay the bright-field images with the fluorescent RFP images.
33. Proceed to the “IdentifyPrimaryObject” section. This is the most important section as it is the step that identifies the DA neurons based on the assigned parameters.

***Note:*** The intensity threshold determines whether each pixel will be considered foreground or background. There are four automatic detection algorithms (otsu, minimum cross entropy, robust background, and measurement) or the parameters can also be set manually. In our pipeline we introduced the manual model but make sure to try different parameters in “test mode” as some threshold strategies may work better with your experiment. The CellProfiler manual also has detailed explanations for each automatic algorithm https://cellprofiler-manual.s3.amazonaws.com/CellProfiler-4.2.6/index.html.

34. Proceed to the “MeasureObjectIntensity” tab to quantify the fluorescent intensity of DA neurons. Export the quantified intensity values using the “ExportToSpreadsheet” command and save the colorized neurons to ensure that correct neuron diameter reference ranges are set.

**Figure 4:**
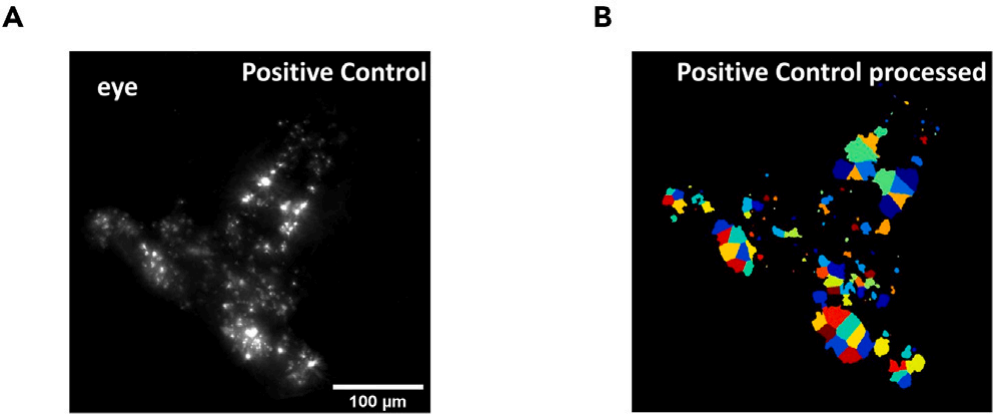
DA neuron isolation and fluorescence intensity analysis Example steps in the CellProfiler pipeline that highlights the identification of TH neurons based on the pixel size. (A) Using the 20x bright field image to overlay the fluorescent image to automatically detect the eyes for removal of autofluorescence. (B) The “IdentifyPrimaryObjects” command with the global threshold strategy configurations correctly capture the neurons that are later used to quantify intensity.

***Note:*** The example of image outputs and the corresponding quantification of object identified outputs are shown in Figure 4.

35. For each 96 well plate analysis, normalize the DA neuron intensity to the positive control and do a student-t test between the positive and negative control.

***Note:*** A video going through the statistical analysis of the spreadsheet output can be found in the data repository https://github.com/happyiowa/screening-protocol.

## Expected outcomes

This protocol was designed as a routine candidate compound screening method spanning the duration of 1 week. This allows for proper planning of experiments, raising larvae, and conducting analysis routinely to scan a large library of compounds *in vivo* for hit identification. If the exact concentrations of MTZ or CBE are used, and the 24 h incubation time is carried out as listed in the protocol, the expected DA neuron intensity loss during the 5 dpf to 6 dpf incubation period should be ∼65% in the negative control. Higher concentrations can result in more significant ablation and can be suitable for some studies. The dose response and rationale for the MTZ or CBE concentration and treatment duration can be found in our previous work.^4^ If there is indeed a candidate compound, the pre-treatment to post-treatment DA neuron intensity will result in a value in between the positive control (0.2% DMSO) and negative control (MTZ or CBE treatment). With a sample size of n = 10 to 12 depending on the successful embedding and imaging, this will be sufficient to conduct a student’s t-test between the negative control and the candidate neuroprotective compound-treated group to determine significance.

## Quantification and statistical analysis

**Figure 5:**
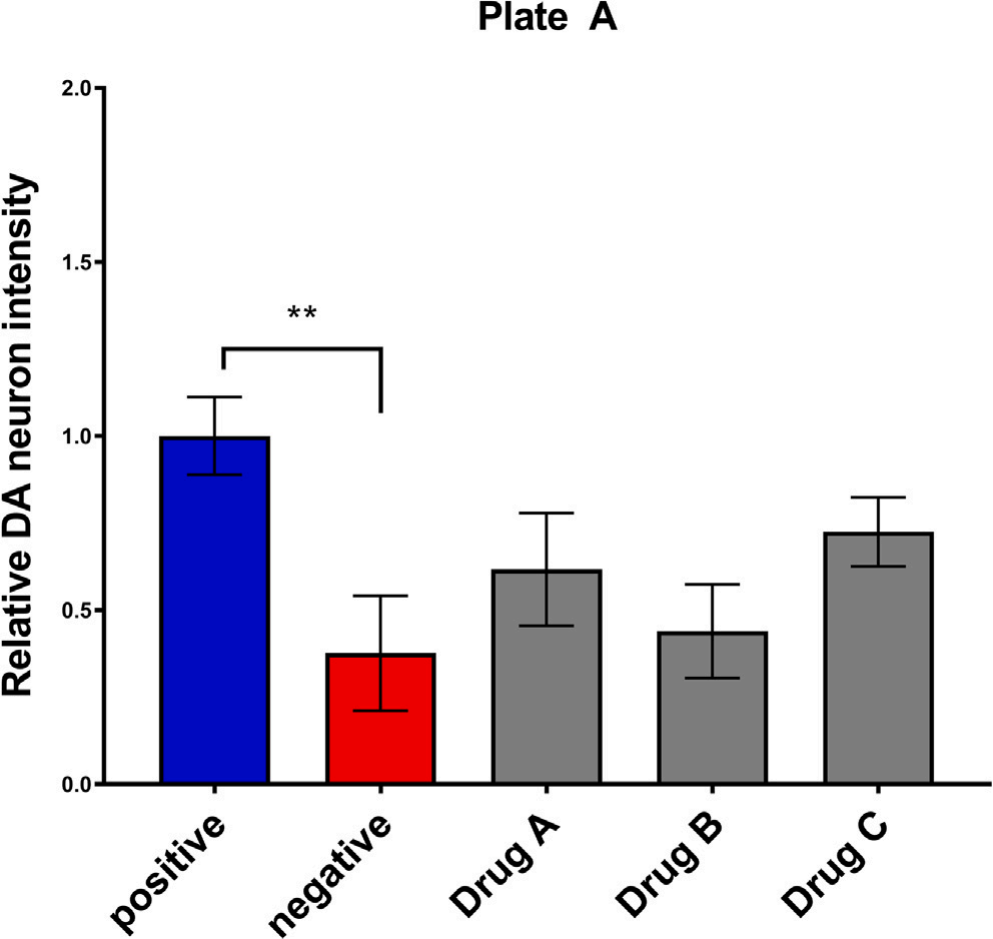
Bar graph visualization of the screening plate analysis A t-test between the positive control (blue) and negative control (red) shows significant DA neuron ablation. The difference between the negative control and the different treatment groups (Drug A, B, and C) should be assessed to determine the significance of a hit candidate.

After running the CellProfiler pipeline for image analysis, the “ExportToSpreadsheet” command will output the .xlsx file as shown in the example Table 1. The “Intensity_TotalIntensity” is the variable that reflects the total fluorescence of the isolated DA neurons. The ratio between the post-treatment and pre-treatment is used to indicate the relative DA neuron intensity. To remove batch effects and minimize variability across different experimental time points, a normalization process is done using the positive control as shown in Table 2. The positive untreated control is set to an average of 1 and the coefficient is used to normalize across all treatment groups including the negative control. The final example figure for a single plate experiment is shown in Figure 5.

**Table 1:** Example raw data output after CellProfiler analysis

FileName_fluorescent	Area_fluorescent	Intensity_TotalIntensity Neuron_before	Intensity_TotalIntensity Neuron_after
A - 03(fld 1 wv Green - dsRed).tif	2048	11765.25	11456.949
A - 04(fld 1 wv Green - dsRed).tif	2048	5455.213	5831.278
A - 05(fld 1 wv Green - dsRed).tif	2048	14997.23	15805.982
A - 06(fld 1 wv Green - dsRed).tif	2048	16545.31	22096.875
B - 03(fld 1 wv Green - dsRed).tif	2048	6432.72	2179.4119
B - 04(fld 1 wv Green - dsRed).tif	2048	13021.84	8220.154
B - 05(fld 1 wv Green - dsRed).tif	2048	7422.13	3103.3438
B - 06(fld 1 wv Green - dsRed).tif	2048	1543.123	902.2307

The “ExportToSpreadsheet” command will output an excel file containing the total area and intensity of the isolated DA neurons.

**Table 2:** Normalization of data using the positive control

Positive control	Negative control	Drug A	Drug B	Drug C
0.687116793	0.792863215	1.333559369	0.01944995	0.95719836
0.221625806	0.022210929	0.587523018	0.005047256	0.273325073
1.304585132	0.015277484	0.003225738	0.571162806	0.941450358
0.939888901	0.676811433	0.84051444	0.033335807	1.069287261
0.993100861	0.031435902	0.228420235	0.302216184	0.673776433
0.973153852	0.632155973	0.992910936	1.446875152	1.073795277
0.927616867	0.033372705	0.965452393	0.372308743	0.669313588
1.298070359	0.000191622	0.054255174	0.570935081	0.241047608
1.23280951	0.008499701	0.000552455	0.626931393	0.899461655
1.42203192	1.545933566	1.164896692	0.441586879	0.452460786

Example screening plate showing the normalization of fluorescent intensity to the positive control.

## Limitations

The screening assay is based on the 5 to 6 dpf larvae which is not representative of the time point of neurodegeneration given that neurodegenerative diseases occur in later stages of life for humans.^5^ Also, the chemical ablation method with CBE or MTZ do not recapitulate the exact etiology or progression of Parkinson’s Disease (PD).^6^^,^^7^ However, our previous work has shown that the NTR-MTZ model and CBE model have mechanistic relevance to PD in the context of mitochondrial dysfunction.^1^ Also, the CBE treatment has been widely used to induce Gaucher’s disease, an autosomal recessive disorder highly linked to PD.^8^^,^^9^

The protocol utilizes a 20x objective lens, which requires precise positioning of the larvae. There are lower magnification lenses such as 4x or 10x objectives which can be more forgiving and capture a larger area of the well, but it will also come with the compromise of lower resolution when doing the data analysis.

As the neuron image analysis with CellProfiler and ImageJ is based on the size of the neuron and intensity, it is prone to capture neurons or particles that are not DA neurons. Upon MTZ and CBE treatment, it is possible that fluorescent puncta remain. Without further validation, our assay is limited on whether those damaged neurons remain functional. It is important to correctly use the sizing tool to ensure those debris are not falsely captured during the quantification.

## Troubleshooting

### Problem 1

Hard to position larvae in the dorsal down position in the 96-well plates (related to step 7 in the agarose embedding in 96 well plate section) .

### Potential solution

**Figure 6:**
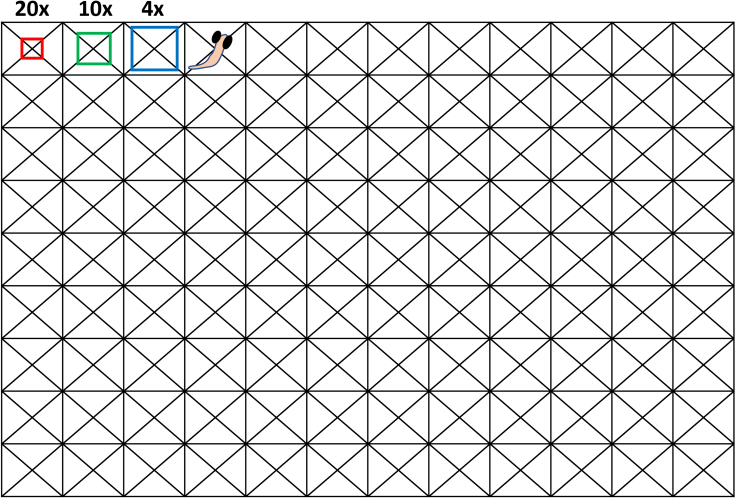
Template for 96-well plate positioning To assist with the proper alignment and embedding of the dorsal down positioning of the larvae, a custom created template is printed and placed in the bottom of the plate. The red, green, and blue squares represent 20x, 10x and 4x imaging area that the IN Cell 6000 Analyzer can cover. The template is available in the repository (Specific for the Greiner μClear plates).

• Try not to position the larvae immediately after placing it in the well. Allowing time for agarose to be slightly solidified helps.
• Make sure to accurately measure out the agarose needed. If 1.2% is difficult, you can also try increasing it slightly more up to 1.5%. However, agarose concentrations beyond 1.5% has shown to be stressful for larvae during the 24-h incubation period.
• The general field of view for each objective lens is depicted in Figure 6. To assist with accurate positioning, create a template that clearly indicates the center of the well and use that as a marker to align all embryos in the general position.

### Problem 2

Identifying DA neurons incorrectly in the image analysis software (referring to step 10 in the image analysis methods).

### Potential solution

• The size of the DA neurons in the diencephalon region can be slightly different for each imaging session. This could result in the CellProfiler not being able to capture the correct neurons, either overcounting or undercounting which can result in inaccurate quantification.
• For each plate analyzed by the CellProfiler software, do a test run on a few positive control samples, adjusting the particle radius “IdentifyPrimaryObjects”.

### Problem 3

No significant DA neuron damage in negative control.

### Potential solution

• The MTZ and CBE should ideally be prepared fresh for each screening as it is important to ensure that there is a significant difference between the positive and negative control. The 9 mM MTZ was chosen based on the consistent ablation of approximately 50% of the DA neurons.
• Do not reuse leftover MTZ stock solution after performing a batch of plates.

### Problem 4

Out of focus images (referring to step 3 of the Confocal Imaging and compound treatment methods).

### Potential solution

• Every embedded plate will have slightly different focus and z-stack. Always make sure to observe several example plates before automating the capture. Since the “max intensity” projection is used, it is okay to have a slightly thicker stack of up to ∼150 μm to ensure all diencephalon regions are captured across the plates.

## Resource availability

### Lead contact

Further information and requests for resources and reagents should be directed to and will be fulfilled by the lead contact, Su Guo (su.guo@ucsf.edu).

### Technical contact

Technical questions on executing this protocol should be directed to and will be answered by the technical contact, Gha-hyun Jeffrey Kim (happyiowa@gmail.com).

### Materials availability

This study did not generate new unique reagents.

### Data and code availability

The IN Cell Analyzer acquisition protocols and custom generated pipelines for DA neuron analysis are all available in https://github.com/happyiowa/screening-protocol.

## References

[1] Kim G.-H.J., Mo H., Liu H., Wu Z., Chen S., Zheng J., Zhao X., Nucum D., Shortland J., Peng L. (2021). A zebrafish screen reveals Renin-angiotensin system inhibitors as neuroprotective via mitochondrial restoration in dopamine neurons. Elife.

[2] Westerfield M. (2007).

[3] Stirling D.R., Swain-Bowden M.J., Lucas A.M., Carpenter A.E., Cimini B.A., Goodman A. (2021). CellProfiler 4: improvements in speed, utility and usability. BMC Bioinf..

[4] Kim G.H.J., Mo H., Liu H., Okorie M., Chen S., Zheng J., Li H., Arkin M., Huang B., Guo S. (2022). In Vivo Dopamine Neuron Imaging-Based Small Molecule Screen Identifies Novel Neuroprotective Compounds and Targets. Front. Pharmacol..

[5] Armstrong M.J., Okun M.S. (2020). Diagnosis and Treatment of Parkinson Disease: A Review. JAMA.

[6] Haddad F., Sawalha M., Khawaja Y., Najjar A., Karaman R. (2017). Dopamine and Levodopa Prodrugs for the Treatment of Parkinson’s Disease. Molecules.

[7] Exner N., Lutz A.K., Haass C., Winklhofer K.F. (2012). Mitochondrial dysfunction in Parkinson′s disease: Molecular mechanisms and pathophysiological consequences. EMBO J..

[8] Riboldi G.M., Di Fonzo A.B. (2019). GBA, Gaucher Disease, and Parkinson’s Disease: From Genetic to Clinic to New Therapeutic Approaches. Cells.

[9] Niederkofler V., Auer E., Amschl D., Neddens J., Hutter-Paier B. (2019). CBE treatment of alpha-synuclein over-expressing and wildtype mice models Gaucher disease pathology. Mol. Genet. Metabol..

